# Prognostic significance of tumor deposits in radically resected gastric cancer: a retrospective study of a cohort of 1915 Chinese individuals

**DOI:** 10.1186/s12957-022-02773-1

**Published:** 2022-09-23

**Authors:** Menglong Zhou, Wang Yang, Wei Zou, Jianing Yang, Changming Zhou, Zhiyuan Zhang, Yaqi Wang, Jing Zhang, Yan Wang, Guichao Li, Zhen Zhang, Fan Xia

**Affiliations:** 1 Department of Radiation Oncology, Fudan University Shanghai Cancer Center, 270 Dong’an Road, Shanghai, 200032 People’s Republic of China; 2 Department of Oncology, Shanghai Medical College, Fudan University, Shanghai, 200032 People’s Republic of China; 3 Shanghai Key Laboratory of Radiation Oncology, Shanghai, 200032 People’s Republic of China; 4 Department of Cancer Prevention, Fudan University Shanghai Cancer Center, Shanghai, 200032 People’s Republic of China

**Keywords:** Gastric cancer, Tumor deposit, Prognosis, Propensity score

## Abstract

**Background:**

Tumor deposits (TDs) have been identified as an independent prognostic factor in gastric cancer (GC). However, the associated clinicopathological factors and how to simply and reasonably incorporate TD into the TNM staging system remain undetermined. The aim of the current study was therefore to assess the significance of TD among radically resected GC patients.

**Methods:**

We retrospectively reviewed 1915 patients undergoing radical resection between 2007 and 2012. The patients were classified into two groups according to TD status (absent vs. present), and the clinicopathologic characteristics, DFS, and OS were compared. Associations of TD presence with other clinicopathologic factors were evaluated by logistic regression analysis. Univariate and multivariate Cox regression analyses were performed to determine the prognostic factors for DFS and OS in the primary cohort. Propensity score matching (PSM) was performed to reduce the possibility of selection bias according to the presence of TD. External validation of previously proposed modified staging systems incorporating TD was conducted.

**Results:**

The detection rate of TD was 10.5% (201/1915). The presence of TD was significantly related to unfavorable clinicopathologic variables, including advanced T and N categories. According to the multivariate Cox regression analysis, the presence of TD was identified as an independent prognostic factor for DFS and OS in the primary cohort (both *P* < 0.001). In the after-PSM cohort, TD presence also significantly shortened DFS and OS. In the external validation, one system that incorporated TD into the pTNM stage had the best performance.

**Conclusions:**

The presence of TD was significantly associated with poor survival in radically resected GC patients. The incorporation of TD into the TNM staging system can further improve the predictive capability. A multicenter cohort with a large sample size is needed to determine the appropriate method of incorporation.

**Supplementary Information:**

The online version contains supplementary material available at 10.1186/s12957-022-02773-1.

## Introduction

According to the latest epidemiological data, gastric cancer (GC) ranks fifth and fourth in terms of the estimated number of new cases and deaths worldwide, respectively [1]. TNM stage is the most commonly used parameter for determining prognosis and for treatment decision-making. Apart from TNM stage, there are other common clinicopathological factors significantly related to prognosis, such as lymphovascular invasion (LVI), perineural invasion (PNI), and tumor grade, which have also been identified in previous studies [2–5].

TD, as a typical histopathological feature of colorectal tumors, was first proposed by Gabriel W.B. as early as 1935 [6]. TDs are tumor-like nodular masses in addition to the primary tumor that are located in the fat tissue of the mesocolon and mesorectum. The role of TD in colorectal cancer has been widely studied [7]. Eventually, the association of poor prognosis with TD in colorectal cancer was confirmed [8–10], and TD status was included in the N1c category of colorectal cancer in the 7th edition of the AJCC staging system [11]. In addition, studies have shown that TD also exists in other cancers, such as breast cancer [12], thyroid cancer [13], lung cancer [14], gastric cancer (GC) [15–17], and pancreatic carcinoma [18].

In gastric cancer, with improvements in surgical and pathological detection technology, the number of detected TDs is gradually increasing, and its role in the staging and prognosis of GC has increasingly become a research focus. First, the presence of TD has been identified as an independent prognostic factor for GC by most previous studies, but the results regarding the clinicopathological factors associated with its presence have been inconclusive [16, 17, 19–28]. Second, whether there is a place for TD in staging and how to simply and reasonably incorporate TDs into the TNM staging system remain undetermined. TDs are likely to be considered metastatic lymph nodes [21, 26, 28], but several studies have indicated that TD should be regarded as serosal invasion [16, 23]. At present, the Japanese gastric cancer treatment guidelines recommend that each TD found in the lymphatic drainage area of the primary tumor should be included in the N category as a metastatic lymph node [29]. However, in the eighth edition of the American Joint Committee on Cancer (AJCC) GC staging system, TD first existed as one of the nineteen registry data collection variables, without mention of the role it may play in prognosis or its incorporation into staging [30]. Therefore, more studies are warranted to clarify the aforementioned aspects.

The current study retrospectively enrolled 1915 patients with resectable GC who underwent radical surgery in our center with the aim of comprehensively evaluating the effect of TD on resectable GC patients, including its association with clinicopathologic factors and its influence on prognosis. In addition, this study summarized and evaluated the existing methods of incorporating TD into the TNM staging system.

## Materials and methods

### Patient population

A cohort of 1915 GC patients who underwent radical resection between January 2007 and December 2012 at Fudan University Shanghai Cancer Center (FUSCC) was retrospectively identified. Patients were eligible if they met the following criteria: (1) histopathologically confirmed gastric or gastroesophageal junction adenocarcinoma; (2) no evidence of distant metastasis or peritoneal seeding on preoperative staging; (3) R0 resection with at least D1 lymphadenectomy; and (4) no preoperative chemotherapy or radiotherapy. The patients’ clinicopathologic and therapeutic factors were retrospectively collected. The current study was approved by the medical ethics committee of FUSCC, and the study was conducted in accordance with the Declaration of Helsinki.

### Treatment delivery

Peritoneal washings were not routinely performed during the study period. Adjuvant treatment including chemotherapy (ChT) or ChT plus concurrent chemoradiotherapy (CRT) was recommended for all patients with stage II–III disease. The ChT regimens included single-agent fluoropyrimidines (tegafur gimeracil oteracil potassium capsules (S-1) or capecitabine), dual drug combinations (fluoropyrimidine plus platinum) or three drug combinations (fluoropyrimidine, platinum plus epirubicin, or taxanes).

Radiotherapy was given with 6 MV photons using either three-dimensional CRT or intensity-modulated radiation therapy (IMRT). Patients were treated with 25 to 28 fractions of 1.8 Gy for a total dose of 45 to 50.4 Gy (5 fractions/week). The clinical target volume (CTV) encompassed the preoperative tumor extension, tumor bed, anastomosis site, and regional draining LNs. The planning target volume (PTV) margin was 0.5 to 1.0 cm considering the individual uncertainties. The remnant stomach wsa not routinely included within the radiation field. Concurrent ChT regimens included (1) a continuous intravenous infusion of 225 mg/m^2^ of 5-fluorouracil (5-FU) for 120 h each week and (2) 825 mg/m^2^ of capecitabine twice daily from day 1 to 5 weekly or S-1 30 mg/m^2^ twice daily from day 1 to 5 weekly.

### Follow-up

All patients were followed-up every 3 months for the first 2 years, then every 6 months until the fifth year, and yearly thereafter. Follow-up examinations included a complete history and physical examination, measurement of serum tumor biomarkers, CT scans of the chest, abdomen and pelvis each time, and endoscopy each year. Disease-free survival (DFS) was defined as the time from surgery to death, locoregional recurrence, or distant recurrence. Overall survival (OS) was defined as the interval from surgery to date of death from any cause or date of the most recent follow-up. Patients for whom none of these events were recorded were censored at the date of their last known contact. The median follow-up time for all the patients was 93.7 months (IQR 73.7–112.9 months).

### Pathology and definition of TD

The histological sections of tumor specimens were reviewed independently by two pathologists, and disagreements were confirmed by a third pathologist. The tumor was classified according to the 8th edition AJCC staging system for GC. TDs are defined and evaluated as discrete foci of cancer cells found in the perigastric fat or adjacent ligament away from the primary lesion but within the area of locoregional lymph node stations. No identifiable lymph node tissue or vascular or neural structure should be found. The shape, contour and size of the deposits are not assessed in these designations.

### Statistical analysis

Comparison of the clinicopathological characteristics between the TD-absent and TD-present groups was performed. Data are summarized as the mean (± standard deviation) or the median (with range) for continuous variables and numbers (percentages) for categorical variables. Continuous data were compared using the t-test/Wilcoxon rank sum test, whereas categorical data were analyzed using the chi-square test. The standardized mean difference (SMD) was reported to assess the balance of covariates between the two groups. Univariate and multivariate models using logistic regression were conducted to assess the relationship between TD status and other clinicopathological characteristics.

DFS and OS were calculated using the Kaplan-Meier method, and the log-rank test was employed to determine the significance. Associations between clinicopathologic features and survival was assessed with univariate analysis. The potentially relevant factors obtained from the univariate analysis were assessed in the multivariate model using Cox regression. Hazard ratios (HR) and 95% confidence intervals (CI) were calculated.

In the logistic and Cox regression analyses, age and tumor size were treated as categorical variables and dichotomized at the cohort median age of 58 years and tumor diameter of 3.2 cm. The number of retrieved lymph nodes was also treated as a categorical variable and dichotomized at the accepted cutoff of 15.

To reduce selection bias, a 1:3 propensity score-matching analysis was performed between the TD-absent and TD-present groups. Propensity scores were estimated using a logistic regression model and the following covariates: sex, age, tumor location, tumor size, histologic grade, vascular emboli, lymphatic and perineural invasion (VELIPI), T category, N category, number of retrieved lymph nodes, and adjuvant treatment. Using these propensity scores, patients with TDs (TD-present group) were individually matched to patients without TDs (TD-absent group).

The predictive abilities of the TNM staging system and other modified systems were evaluated by the *χ*^2^ value, the area under the receiver operating characteristic curve (AUC), Harrell's concordance index (C-index) and Akaike information criterion (AIC). Larger *χ*^2^, AUC, and C-index values and a smaller AIC value indicate that the system has a better discriminative ability.

A two-sided significance level of 0.05 was applied. All statistical analyses were performed using the R statistical software package (version 4.2.1; R Project for Statistical Computing, Vienna, Austria).

## Results

###  Clinicopathologic characteristics

In total, 1915 patients were enrolled in this cohort, and TD was present in 201 patients. Thus, the detection rate was 10.5%. The clinical and pathologic characteristics of the cohort were summarized according to TD status (Table 1). Of the 1915 patients, 1333 were men (69.6%), and 582 were women (30.4%). with a median age of 58 years (range, 19 to 84 years). The mean number of lymph nodes dissected per patient was 23.4 ± 8.4. Of these patients, 71.9% (1376/1915) received adjuvant treatment, including 1242 patients who received adjuvant ChT and 134 patients who received adjuvant CRT.

**Table 1:** Comparison of clinicopathologic features between TD-absent and TD-present gastric cancer patients before and after PSM

Variables	Before PSM						After PSM					
	TD-absent		TD-present		*P* value	SMD	TD-absent		TD-present		*P* value	SMD
	*n*	%	*n*	%			*n*	%	*n*	%		
Overall	1714		201				491		191			
Sex												
Male	1184	69.1	149	74.1	0.164	0.112	355	72.3	140	73.3	0.868	0.022
Female	530	30.9	52	25.9			136	27.7	51	26.7		
Age (years)												
< 58	917	53.5	78	38.8	< 0.001	0.298	205	41.8	77	40.3	0.798	0.029
≥ 58	797	46.5	123	61.2			286	58.2	114	59.7		
Mean (SD)	56.9 ± 11.1		61.0 ± 10.6				59.9 ± 10.6		60.7 ± 10.6			
Median (range)	58 (19–82)		62 (33–84)				61 (28–82)		62 (33–84)			
Tumor location												
GEJ + Upper 1/3	431	25.1	68	33.8	0.013	0.217	163	33.2	65	34.0	0.964	0.023
Middle 1/3	417	24.3	51	25.4			122	24.8	48	25.1		
Lower 1/3	866	50.5	82	40.8			206	42.0	78	40.8		
Tumor size												
<3.2 cm	922	53.8	45	22.4	< 0.001	0.683	145	29.5	45	23.6	0.142	0.136
≥ 3.2 cm	792	46.2	156	77.6			346	70.5	146	76.4		
Mean (SD)	3.6 ± 2.1		5.1 ± 2.3				4.7 ± 2.3		4.9 ± 2.1			
Median (range)	3.0 (0.3–16.0)		4.5 (0.8–14.0)				4.5 (0.5–14)		6.0 (0.8–12)			
Histologic grade												
Well-moderately	325	19.0	29	14.4	0.141	0.122	68	13.8	28	14.7	0.880	0.023
Poorly	1389	81.0	172	85.6			423	86.2	163	85.3		
T category												
T1	521	30.4	2	1.0	< 0.001	1.131	6	1.2	2	1.0	0.508	0.148
T2	255	14.9	11	5.5			35	7.1	11	5.8		
T3	187	10.9	14	7.0			42	8.6	14	7.3		
T4a	731	42.6	158	78.6			393	80.0	153	80.1		
T4b	20	1.2	16	8.0			15	3.1	11	5.8		
N category												
N0	790	46.1	16	8.0	< 0.001	1.023	50	10.2	16	8.4	0.846	0.100
N1	308	18.0	36	17.9			97	19.8	36	18.8		
N2	280	16.3	50	24.9			132	26.9	49	25.7		
N3a	259	15.1	74	36.8			164	33.4	67	35.1		
N3b	77	4.5	25	12.4								
TNM stage												
I	620	36.2	3	1.5	< 0.001	1.209	16	3.3	3	1.6	0.365	0.128
II	398	23.2	22	10.9			66	13.4	22	11.5		
III	696	40.6	176	87.6			409	83.3	166	86.9		
VELIPI												
Negative	996	58.1	54	26.9	< 0.001	0.666	147	29.9	54	28.3	0.738	0.037
Positive	718	41.9	147	73.1			344	70.1	137	71.7		
No. of retrieved LNs												
< 15	331	19.3	36	17.9	0.702	0.036	86	17.5	33	17.3	1.000	0.006
≥ 15	1383	80.7	165	82.1			405	82.5	158	82.7		
Mean (SD)	21.3 ± 8.4		21.9 ± 8.4				21.9 ± 8.3		22.0 ± 8.5			
Median (range)	20 (2–74)		20 (8–62)				20 (5–55)		20 (8–62)			
Adjuvant treatment												
No	501	29.2	38	18.9	< 0.001	0.388	66	13.4	32	16.8	0.812	0.106
Single drug	304	17.7	40	19.9			104	21.2	38	19.9		
Double drugs	625	36.5	63	31.3			177	36.0	63	33.0		
Triple drugs	167	9.7	43	21.4			101	20.6	41	21.5		
CRT	117	6.8	17	8.5			43	8.8	17	8.9		

*Abbreviations*: *CI* confidence interval, *GEJ* gastroesophageal junction, *LNs* lymph nodes, *PSM* propensity score matching, *SD* standard difference, *SMD* standardized mean difference, *TD* tumor deposit, *VELIPI* vascular emboli, lymphatic, and perineural invasion

Among the 201 TD-present patients, a total of 329 TDs were detected, ranging from 1 to 12 TDs. The median and average numbers were 1 and 1.64, respectively. A total of 132 patients had 1 TD, 43 patients had 2 TDs, and 26 patients had ≥ 3 TDs. The common distribution areas were the lesser curvature omentum (*n* = 191), greater curvature omentum (*n* = 80), greater omentum (*n* = 42), and other areas (*n* = 16) (Supplementary Table 1).

The presence of TD was significantly associated with older age, distal tumor location, larger tumor size, advanced T category, advanced N category, and VELIPI. In multivariate logistic regression, older age, larger tumor size, advanced T category, and advanced N category were recognized as independent risk factors for the presence of TD (Table 2).

**Table 2:** Association of TD presence with clinicopathologic characteristics

Variables	Univariate logistic regression			Multivariate logistic regression		
	Crude OR	95% CI	*P* value	Adjusted OR	95% CI	*P* value
Sex						
Male	Ref.					
Female	0.78	0.56–1.08	0.142			
Age (years)						
< 58	Ref.			Ref.		
≥ 58	1.81	1.35–2.46	< 0.001	1.48	1.07–2.05	0.018
Tumor location						
GEJ + Upper 1/3	Ref.			Ref.		
Middle 1/3	0.67	0.47–0.93	0.019	1.08	0.70–1.64	0.732
Lower 1/3	0.55	0.41–0.73	< 0.001	1.07	0.74–1.56	0.714
Tumor size						
< 3.2 cm	Ref.			Ref.		
≥ 3.2 cm	4.04	2.88–5.76	< 0.001	1.60	1.10–2.35	0.016
Histologic grade						
Well-moderately	Ref.					
Poorly	1.39	0.93–2.13	0.119			
T category						
T1	Ref.			Ref.		
T2	11.24	2.99–72.96	0.002	6.34	1.65–41.65	0.018
T3	19.50	5.38–124.96	< 0.001	6.96	1.82–45.84	0.013
T4a	56.31	17.89–341.41	< 0.001	17.78	4.86–105.80	< 0.001
T4b	208.40	54.60–1377.01	< 0.001	51.88	12.33–360.701	< 0.001
N category						
N0	Ref.			Ref.		
N1	5.77	3.21–10.83	< 0.001	3.10	1.68–5.94	< 0.001
N2	8.82	5.05–16.21	< 0.001	3.56	1.98–6.73	< 0.001
N3a	14.11	8.29–25.49	< 0.001	4.99	2.83–9.31	< 0.001
N3b	16.03	8.28–31.89	< 0.001	5.03	2.50–10.41	< 0.001
VELIPI						
Negative	Ref.			Ref.		
Positive	3.78	2.74–5.27	< 0.001	1.13	0.79–1.65	0.508

*Abbreviations*: *CI* confidence interval, *GEJ* gastroesophageal junction, *VELIPI* vascular emboli, lymphatic, and perineural invasion, *TD* tumor deposit, *OR* odds ratio, *Ref*. reference

### Survival analysis

According to the univariate analysis shown in Table 3, the following 10 clinicopathologic characteristics were demonstrated to be significantly associated with DFS and OS in the primary cohort: age (< 58 vs. ≥ 58, years), tumor location, tumor size (< 3.2 vs. ≥ 3.2, cm), histologic grade, T category, N category, VELIPI, adjuvant treatment, and TD status. After multivariate Cox proportional hazards model analysis, T category, N category, VELIPI, adjuvant treatment, and TD status remained independent prognostic factors for DFS, and age, T category, N category, adjuvant treatment, and TD status remained independent prognostic factors for OS (Table 4).

**Table 3:** Univariate analyses for DFS and OS of GC patients

Variables	*n*	Disease-free survival			Overall survival		
		HR	95% CI	*P* value	HR	95% CI	*P* value
Overall	1915						
Sex							
Male	1333	Ref.			Ref.		
Female	582	0.93	0.77–1.11	0.400	0.87	0.72–1.06	0.161
Age (years)							
< 58	995	Ref.			Ref.		
≥ 58	920	1.40	1.19–1.65	< 0.001	1.65	1.39–1.96	< 0.001
Tumor location							
GEJ + Upper 1/3	499	Ref.			Ref.		
Middle 1/3	468	0.82	0.66–1.01	0.063	0.81	0.65–1.00	0.053
Lower 1/3	948	0.54	0.44–0.66	< 0.001	0.55	0.45–0.67	< 0.001
Tumor size							
< 3.2 cm	967	Ref.			Ref.		
≥ 3.2 cm	948	2.93	2.45–3.51	< 0.001	3.05	2.53–3.68	< 0.001
Histologic grade							
Well-moderately	354	Ref.			Ref.		
Poorly	1516	1.62	1.27–2.06	< 0.001	1.51	1.19–1.92	< 0.001
T category							
T1	523	Ref.			Ref.		
T2	266	2.49	1.53–4.06	< 0.001	2.62	1.56–4.41	< 0.001
T3	201	5.68	3.60–8.95	< 0.001	6.11	3.77–9.92	< 0.001
T4a	889	11.93	8.18–17.38	< 0.001	13.05	8.71–19.56	< 0.001
T4b	36	22.94	13.34–39.45	< 0.001	28.20	15.97–49.77	< 0.001
N category							
N0	806	Ref.			Ref.		
N1	344	2.49	1.81–3.43	< 0.001	2.68	1.94–3.71	< 0.001
N2	330	5.23	3.95–6.94	< 0.001	5.08	3.79–6.82	< 0.001
N3a	333	10.23	7.86–13.32	< 0.001	10.34	7.87–13.58	< 0.001
N3b	102	16.90	12.30–23.22	< 0.001	18.88	13.61–26.20	< 0.001
VELIPI							
Negative	1050	Ref.			Ref.		
Positive	865	3.72	3.10–4.46	< 0.001	3.43	2.86–4.12	< 0.001
No. of retrieved LNs							
< 15	367	Ref.			Ref.		
≥ 15	1548	1.22	0.98–1.51	0.078	1.17	0.93–1.45	0.175
Adjuvant treatment							
No	539	Ref.			Ref.		
Single drug	344	1.57	1.17–2.11	0.003	1.54	1.13–2.10	0.006
Double drugs	688	2.13	1.66–2.71	< 0.001	2.27	1.76–2.92	< 0.001
Triple drugs	210	3.24	2.44–4.31	< 0.001	2.98	2.21–4.02	< 0.001
CRT	134	3.79	2.77–5.20	< 0.001	3.58	2.58–4.97	< 0.001
TD							
Absent	1714	Ref.			Ref.		
Present	201	4.14	3.42–5.02	< 0.001	4.45	3.66–5.42	< 0.001

*Abbreviations*: *CI* confidence interval, *DFS* disease-free survival, *GC* gastric cancer, *GEJ*, gastroesophageal junction, *HR* hazard ratio, *LNs* lymph nodes, *OS* overall survival, *PSM* propensity score matching, *Ref.* reference, *TD* tumor deposit, *VELIPI* vascular emboli, lymphatic, and perineural invasion

**Table 4:** Multivariate Cox regression analyses for DFS and OS of GC patients

Variables	Disease-free survival			Overall survival		
	HR	95% CI	*P* value	HR	95% CI	*P* value
Age (years)						
< 58	Ref.			Ref.		
≥ 58	1.12	0.94–1.33	0.225	1.34	1.12–1.61	0.002
Tumor location						
GEJ + Upper 1/3	Ref.			Ref.		
Middle 1/3	1.02	0.82–1.27	0.845	1.05	0.84–1.31	0.691
Lower 1/3	0.86	0.70–1.05	0.139	0.88	0.72–1.08	0.230
Tumor size						
< 3.2 cm	Ref.			Ref.		
≥ 3.2 cm	1.17	0.96–1.42	0.122	1.17	0.95–1.43	0.133
Histologic grade						
Well-moderately	Ref.			Ref.		
Poorly	1.12	0.87–1.44	0.382	1.07	0.83–1.38	0.606
T category						
T1	Ref.			Ref.		
T2	1.79	1.07–3.00	0.026	1.94	1.13–3.35	0.017
T3	2.93	1.75–4.92	< 0.001	3.31	1.92–5.71	< 0.001
T4a	4.68	2.92–7.50	< 0.001	5.54	3.36–9.13	< 0.001
T4b	6.68	3.54–12.60	< 0.001	8.99	4.63–17.44	< 0.001
N category						
N0	Ref.			Ref.		
N1	1.61	1.15–2.26	0.006	1.69	1.20–2.39	0.003
N2	2.58	1.88–3.52	< 0.001	2.46	1.78–3.40	<0.001
N3a	4.70	3.46–6.38	< 0.001	4.78	3.49–6.55	< 0.001
N3b	7.18	4.99–10.33	< 0.001	7.97	5.48–11.60	< 0.001
VELIPI						
Negative	Ref.			Ref.		
Positive	1.27	1.03–1.57	0.027	1.11	0.89–1.37	0.357
Adjuvant treatment						
No	Ref.			Ref.		
Single drugs	0.62	0.45–0.84	0.002	0.57	0.41–0.79	0.001
Double drugs	0.55	0.41–0.72	< 0.001	0.56	0.42–0.75	< 0.001
Triple drugs	0.64	0.47–0.88	0.005	0.58	0.42–0.81	0.001
CRT	0.66	0.47–0.94	0.021	0.60	0.42–0.86	0.005
TD						
Absent	Ref.			Ref.		
Present	1.75	1.42–2.14	< 0.001	1.93	1.57–2.38	< 0.001

Abbreviations: *CI* confidence interval, *DFS* disease-free survival, *GC* gastric cancer, *GEJ* gastroesophageal junction, *HR* hazard ratio, *LNs* lymph nodes, *OS* overall survival, *PSM* propensity score matching, *Ref.* reference, *TD* tumor deposit, *VELIPI* vascular emboli, lymphatic, and perineural invasion

**Fig. 1:**
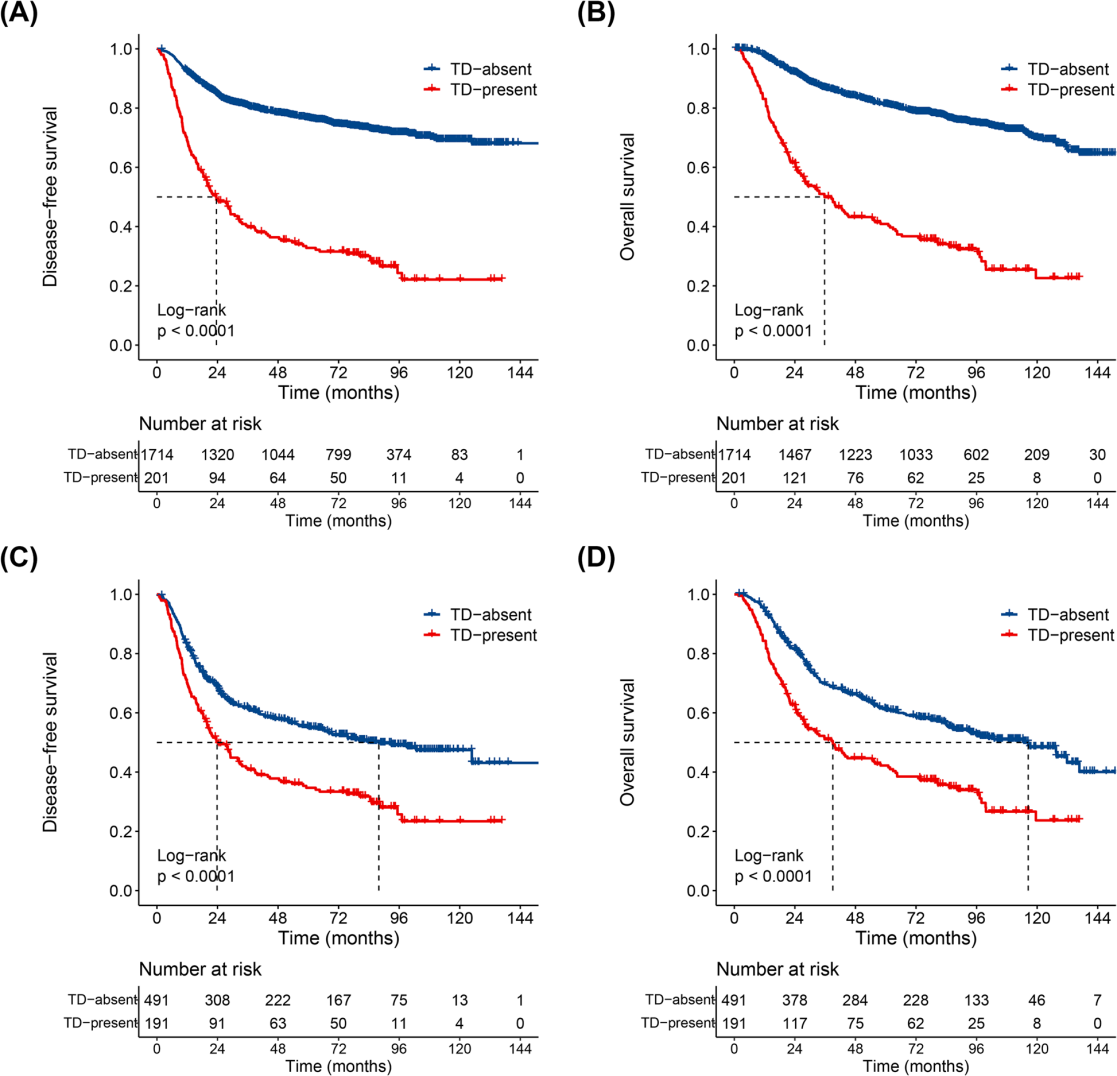
Disease-free survival and overall survival between TD-absent and TD-present patients in the primary cohort (**A**, **B**) and after-PSM cohort (**C**, **D**). Abbreviations: PSM, propensity score matching; TD, tumor deposit

The 3-year DFS and OS rates of all enrolled patients were 76.2% (95% CI 74.3–78.2%) and 82.7% (95% CI 81.0–84.5%), respectively. There was a significant difference in the 3-year DFS rate between patients with TDs and those without (39.8% vs. 80.6%; HR: 1.75, 95% CI 1.42–2.14; *P* < 0.001; Fig. 1A). Additionally, the 3-year OS rate of the TD-present group was significantly lower than that of the TD-absent group (49.9% vs. 86.7%; HR: 1.93, 95% CI 1.57–2.38; *P* < 0.001; Fig. 1B).

In addition to TD status, the influence of TD number on prognosis was evaluated. This continuous variable was transformed into categorical variables by four different cutoff selections; however, no association with prognosis was found regardless of the cutoff selection (Supplementary Table 2).

### Propensity score-matching analysis

After 1:3 PSM, 191 TD-present patients and 491 TD-absent patients were obtained, and no significant differences were identified between the two groups in any of the baseline factors, which was demonstrated by both the *P* value and the SMD (Table 1). In the postmatched dataset, the DFS and OS of the TD-absent group were significantly longer than those of the TD-present group, which is consistent with the results of the survival analysis of the primary cohort. The median DFS was 87.9 months in the TD-absent group and 23.8 months in the TD-present group. The 3-year DFS rate was 61.5% in the TD-absent group and 40.8% in the TD-present group (HR 1.78, 95% CI 1.44–2.21; *P* < 0.001) (Fig. 1C). The 3-year OS rate was 69.9% in the TD-absent group and 51.1% in the TD-present group (HR 1.92, 95% CI 1.54–2.39; *P* < 0.001) (Fig. 1D).

### External validation of the previously proposed modified stage

The current primary cohort was utilized to compare the predictive capabilities of eight existing modified staging systems [16, 20, 21, 23, 26–28, 31] with that of the eighth AJCC TNM staging system (Table 5). Larger *χ*^2^, AUC, and C-index values and a smaller AIC value indicate a better discriminative capability. Accordingly, the system proposed by Gu L. et al. [31] had the best performance. Among these systems, six had a better performance than the TNM staging system, except the system that suggests the presence of TD as T4a, which has a slightly worse performance than the 8th AJCC TNM staging system.

**Table 5:** Comparison of the performance of the TNM staging system and other revised staging systems for GC

Year	Authors	Description	TNM stage edition	*χ*^2^	AUC	95% CI	C-index	95% CI	AIC (f)
2011	Wang W. et al.	Presence of TDs (1–2) as pN3 category. Presence of TDs (≥ 3) as M1 category.	7^a^	609.13	0.837	0.817–0.857	0.798	0.782–0.814	7102.251
2012 2017	Sun Z. et al. Anup S. et al.	Presence of TDs as T4a	7	603.16	0.834	0.814–0.854	0.795	0.779–0.811	7106.225
2013	Lee H.S. et al.	1 TD as 1 positive LN; revised N category	7^b^	612.53	0.832	0.812–0.852	0.793	0.777–0.809	7096.855
2017	AJCC	8th GC’s TNM stage (without TDs)	8	603.77	0.834	0.814–0.855	0.795	0.779–0.811	7105.614
2017	AJCC	1 TD as 1 positive LN (8th GC’s TNM N category)	8	628.90	0.841	0.821–0.861	0.800	0.784–0.816	7080.478
2018	Chen H. et al.	Presence of TDs upstage N stage except for N3b	8	615.94	0.839	0.819–0.859	0.801	0.785–0.817	7093.440
2019	Liang Y. et al.	Presence of TDs upstage N category as follows: N0→mN2; N1→mN2; N2→mN3a. Others unmentioned remained unchanged.	8	630.49	0.841	0.821–0.861	0.800	0.784–0.816	7078.888
2019	Tan J. et al.	Presence of TDs as N3	7^a^	606.83	0.836	0.816–0.857	0.797	0.781–0.813	7102.546
2020	Gu L. et al.	Presence of TDs upstage TNM stage except for IIIC	8	643.57	0.843	0.823–0.864	0.805	0.789–0.821	7065.813

*Abbreviations*: *AIC* Akaike information criterion, *AUC* area under the curve, *CI* confidence interval, *GC* gastric cancer, *LNs* lymph nodes, *TD* tumor deposit

Larger *χ*^2^, AUC, and C–index values and a smaller AIC value indicate that the system has a better discriminative ability

^a^We used the 8th edition of the TNM staging system for GC in our validation, but in these two proposals, N3 was not further divided into N3a or N3b, so we could only use the 7th edition of the TNM staging system for GC

^b^N category was revised

## Discussion

In the current study, we retrospectively analyzed 1915 GC patients who underwent radical gastrectomy, aiming to investigate the role of the presence of TD in GC patients. The presence of TD was associated with unfavorable clinicopathologic factors, reflecting aggressive disease. Moreover, TD was identified as an independent unfavorable indicator of DFS and OS among radically resected GC patients in both the primary cohort and the after-PSM cohort. In addition, this study summarized and evaluated the existing methods of incorporating TD into the TNM staging system.

The incidence of TD in our cohort was 10.5%, which is similar to the previously reported range of the incidence rate (10.6–27.5%) [23, 26]. Moreover, we confirmed that the presence of TD was related to unfavorable clinicopathologic factors, reflecting a more progressive disease. In the primary cohort before PSM, the presence of TD was significantly associated with older age, distal GC, larger tumor size, advanced T category, N category, TNM stage, and VELIPI. Additionally, Lee H.S. et al. reported that TD-present status in the resection specimen was associated with the presence of synchronous distant metastasis [21]. Etoh T. et al. found that TD-present patients were more likely to present with peritoneal seeding at the time of surgery and develop peritoneal metastasis [17].

As TD is associated with unfavorable clinicopathologic factors, it is unsurprising that TD has been confirmed as an independent prognostic factor, together with T and N category, by many previous studies [17]. To estimate the prognostic influence of TD status on GC patients, univariate and multivariate Cox regression analyses were performed in the primary cohort, proving that TD was an independent prognostic factor for DFS and OS. Moreover, an after-PSM cohort was established by 1:3 PSM, and a negative impact of TD on DFS and OS still existed, which was consistent with the Cox regression results.

In addition to TD status (present vs. absent), which most studies evaluated, the evaluable parameters of TD also include the number of TDs and the categorization methods, the patterns of TD and the distribution area of TD. Wang W. et al. [20] transformed the number of TDs into categorical variables (0, 1, 2, and ≥ 3), and a correlation between this parameter and prognosis was found. Similar results were obtained by Sun Z. et al. [16] and Etoh T. et al. [17], but the transformation cutoff values were slightly different (1, 2–3, and > 3 vs. 0, 1–4, and ≥ 5). However, both Anup S. et al. [23] and the current study did not find an association between the number of TDs and prognosis. Lee H.S. et al. [21] classified TD into five types (separate nodular, perivascular, perineural, lymphatic, and endovascular), but no association between TD types and prognosis in GC patients was found. The current study conducted a descriptive statistical analysis of the distribution areas of TD but did not correlate this variable with prognosis because there are various distribution areas of TD, and each patient may have more than one distribution area.

To date, the depth of tumor invasion (T category), nodal status (N category) and distant metastasis (M category), which constitute the TNM stage used for prognostic prediction and guiding treatment, are the three most significant prognostic factors [30]. However, as previous and current studies have shown, the presence of TD is an indicator of poor prognosis in GC patients, and attempts have been made to incorporate TD into the TNM staging system. In this study, we evaluated seven previously presented proposals.

These proposals were suggested based on individual cohorts by comparing the prognosis of patients with TD with that of patients with different T or N categories to achieve the incorporation of TD. First, some proposals incorporated TD into T stage [16, 23]. Sun Z. et al. proposed that TD should be considered a form of serosal invasion (T4a), as they analyzed 2998 GC patients undergoing radical resection and found that no significant difference was observed between the prognosis of TD-present patients in the pT1-4a category and TD-absent patients in the pT4a category [16]. This finding was validated by another study in 2017 [23]. Second, suggestions that TD should be incorporated into the N category in different ways have been proposed by several studies [21, 26, 28, 32]. Kim et al. proposed that each TD should be treated as a positive lymph node, and a new N category was defined [21]. In contrast, Chen H. et al. proposed that the presence of TD should upstage the N category except for N3b [28]. Third, Wang W. et al. considered TD as a significant indicator and suggested incorporating TD into the N3 or M1 staging categories based on the number of retrieved TDs [20]. In addition, Etoh T. et al. suggested that TDs in GC may more closely resemble peritoneal metastasis than lymph node metastasis, but as there were long-term survivors with TD, they should be considered separately from peritoneal disease [17].

In addition to the different methods of integrating TD into the T, N or M categories, the status and number of TDs are another aspect to be considered. Some schemes considered only the status of TD (absent vs. present) [16, 23, 26–28]; some schemes counted one TD as a metastatic lymph node or a metastatic nodule [21]; and some schemes transformed the number of TDs into categorical variables [20]. In addition to the above considerations, most of the schemes did not change the division of the T and N categories, except Lee H.S. et al., who proposed a modified N category [21].

Based on the external validation results of the current dataset, the scheme proposed by Gu L. et al. [31] achieves the best performance. However, how to include TD in TNM staging remains unclear. First, the above seven proposals were presented based on their single-center databases, and external validation data were not provided. Unsurprisingly, the proposed systems performed well in the internal validation. Second, the detection rate of TD in early T or N categories is very low, which is not conducive to evaluating the role of TD. Therefore, the extrapolation of the current existing schemes needs to be further validated. Additionally, a multicenter dataset with a large sample size is needed to verify these schemes or to create a more appropriate proposal.

Although the presence of TD was associated with unfavorable survival in GC patients, the survival rate and multivariate analysis results of our cohort suggest that patients with TD undergoing radical surgery combined with adjuvant therapy can obtain a satisfactory prognosis, indicating that en bloc clearance of adipose connective tissue by D2/R0 surgery is effective. Moreover, for patients with good performance and adherence, adjuvant treatment is highly recommended. Meanwhile, although many studies have confirmed that TD is related to adverse clinicopathologic factors and that TD is an independent prognostic factor, further validations are still needed. Therefore, it is imperative to formulate corresponding standards so that TDs can be appropriately retrieved, examined and recorded.

The first limitation of this study is its retrospective nature. Only the number and anatomic location of the TD were recorded, and there were no data concerning TD patterns. Some variables, such as the gross type and Lauren’s classification of the tumor, were unavailable in some patients. Thus, these variables were not included in the analysis. Second, since this study focused on patients with locally advanced GC, it was not possible to compare the effect of the status and number of TDs on the prognosis of these patients with that of M1 patients. Therefore, this study evaluated only the impact of TD status on the prognosis of patients with different T and N categories. Third, all the patients were from a single center. Whether the results can be extrapolated to other patient groups needs to be further confirmed. Thus, prospective studies with larger sample sizes and more comprehensive information are needed to achieve more convincing conclusions.

## Conclusions

In summary, the presence of TD was associated with unfavorable clinicopathologic factors, reflecting aggressive disease. Moreover, TD was identified as an independent unfavorable indicator of DFS and OS among radically resected GC patients. Incorporating TD into the TNM staging system can further improve the prognostic prediction accuracy, but the appropriate method of incorporation still needs to be explored and verified in prospective cohorts with larger sample sizes and more comprehensive information. In the future, corresponding standards must be formulated so that TDs can be appropriately retrieved, examined and recorded and the significance of TD in GC can be evaluated more comprehensively.

## Supplementary Information

**Additional file 1: Supplementary Table 1.** TD distribution areas and numbers. Abbreviations: TD, tumor deposit.

**Additional file 2: Supplementary Table 2.** Effect of number of TDs on OS in GC patients. Abbreviations: CI, confidence interval; DFS, disease-free survival; GC, gastric cancer; HR, hazard ratio; No., number; OS, overall survival; Pts, patients; Ref., reference; TD, tumor deposit.

## Data Availability

The datasets used during the current study are available from the corresponding author on reasonable request.

## Abbreviations

AIC: Akaike information criterion
AJCC: American Joint Committee on Cancer
AUC: area under the curve
C-index: concordance index
FUSCC: Fudan University Shanghai Cancer Center
GC: gastric cancer
IPTW: inverse probability of treatment weighting
OS: overall survival
PSM: propensity score matching
SMD: standardized mean difference
TD: tumor deposit
VELIPI: Vascular emboli, lymphatic, and perineural invasion
