# Supplementary material for: Prognostic significance of tumor deposits in radically resected gastric cancer: a retrospective study of a cohort of 1915 Chinese individuals
Source: World J Surg Oncol. 2022 Sep 23;20:304. doi: 10.1186/s12957-022-02773-1 (PMC9502614; doi:10.1186/s12957-022-02773-1)
Supplement: Supplementary file 1 — Additional file 1: Supplementary Table 1. TD distribution areas and numbers. Abbreviations: TD, tumor deposit. [file 12957_2022_2773_MOESM1_ESM.docx]

Supplementary Table 1. TD distribution areas and numbers

| Distribution areas | TD numbers |
| --- | --- |
| TD at the lesser curvature omentum | 191 |
| TD at the greater curvature omentum | 80 |
| TD at the greater omentum | 42 |
| TD at the paracardial area | 6 |
| TD along the common hepatic artery | 1 |
| TD in the hepatoduodenal ligament | 4 |
| TD at the pancreas | 2 |
| TD at the infrapyloric area | 3 |
| Total | 329 |

Abbreviations: TD, tumor deposit.
