# Supplementary material for: Prognostic significance of tumor deposits in radically resected gastric cancer: a retrospective study of a cohort of 1915 Chinese individuals
Source: World J Surg Oncol. 2022 Sep 23;20:304. doi: 10.1186/s12957-022-02773-1 (PMC9502614; doi:10.1186/s12957-022-02773-1)
Supplement: Supplementary file 2 — Additional file 2: Supplementary Table 2. Effect of number of TDs on OS in GC patients. Abbreviations: CI, confidence interval; DFS, disease-free survival; GC, gastric cancer; HR, hazard ratio; No., number; OS, overall survival; Pts, patients; Ref., reference; TD, tumor deposit. [file 12957_2022_2773_MOESM2_ESM.docx]

Supplementary Table 2. Effect of the number of TDs on DFS and OS of GC patients.

|  |  | Disease-free survival | | | Overall survival | | |
| --- | --- | --- | --- | --- | --- | --- | --- |
| No. of TDs | No. of pts | HR | 95% CI | *P* value | HR | 95% CI | *P* value |
| Cutoff1 |  |  |  |  |  |  |  |
| 1 | 132 | Ref. |  |  | Ref. |  |  |
| 2 | 43 | 1.02 | 0.67-1.56 | 0.926 | 0.95 | 0.62-1.46 | 0.830 |
| ≥3 | 26 | 1.06 | 0.64-1.73 | 0.829 | 0.96 | 0.57-1.61 | 0.867 |
| Cutoff2 |  |  |  |  |  |  |  |
| 1 | 132 | Ref. |  |  | Ref. |  |  |
| 2-3 | 60 | 1.00 | 0.69-1.45 | 0.991 | 0.93 | 0.64-1.36 | 0.723 |
| ≥4 | 9 | 1.27 | 0.59-2.73 | 0.547 | 1.12 | 0.49-2.56 | 0.789 |
| Cutoff3 |  |  |  |  |  |  |  |
| 1-2 | 175 | Ref. |  |  | Ref. |  |  |
| ≥3 | 26 | 1.05 | 0.65-1.71 | 0.840 | 0.97 | 0.58-1.61 | 0.899 |
| Cutoff4 |  |  |  |  |  |  |  |
| 1-4 | 195 | Ref. |  |  | Ref. |  |  |
| ≥5 | 6 | 1.72 | 0.70-4.22 | 0.233 | 1.46 | 0.54-3.95 | 0.459 |

Abbreviations: CI, confidence interval; DFS, disease-free survival; GC, gastric cancer; HR, hazard ratio; No., number; Obs., observed; OS, overall survival; Pts, patients; Ref., reference; TD, tumor deposit.
